# Anterior Pillars of the Fauces—Etiology and Treatment

**Published:** 1897-08

**Authors:** George T. Carpenter

**Affiliations:** Chicago, Ills.


### Anterior Pillars of the Fauces—Etiology and Treatment.

BY GEORGE T. CARPENTER, M.D., D.D.S , CHICAGO, ILLS.

Abstract of paper read before the Dental Section, American Medical Association, Philadelphia, June, 1897.

It is the dark red, or reddish-blue color, which is a venous stasis of the pillars and uvula, and their shining, sticky, mucous surfaces that I wish to consider. On investigation I find that in a large number of persons visiting my office for dental services, this abnormal condition is present. These parts are easily brought to view by having the patient breathe through the mouth. The patients do not complain of a sore throat but of a fetid breath and somtimes a disagreeable taste in the mouth. The venous stasis of the pillars acts as a sentinel, the etiology of which is not found in the erupting molar—will be found in the tonsil or posterior nares. The tonsil may have little or no inflammatory action and the crypts may or may not be filled with a cheesy exudate, and still their sluggish condition will produce a foul-smelling secretion and absorption of the lymphatics will follow. Also stenosis and chronic catarrhal conditions of the posterior nares will produce a dropping in the pharynx and keep the pillars bathed with a foul secretion which will taint the breath and produce, through absorption, an abnormal appearance of the pillars. Treatment consists in antiseptic gargles of which Borolyptol, either in full strength or diluted, is one of the best.

Also an alkaline and antiseptic wash, as Dobell's solution, used as a gargle or spray for the throat, and spray for the nose. The tonsillar condition may be stimulated with silver nitrate, or the electric cautery may be used to advantage. When catarrhal conditions exist any stenosis should be corrected, and the nares and fauces cleansed every night and morning with an alkaline spray. In addition to this, two or three times a week, iodin solution, two to ten grains to the ounce of water, with iodid of potassium and glycerine will be found advantageous.

